# Preliminary Evidence of Biological and Cognitive Efficacy of Prismatic Adaptation Combined with Cognitive Training on Patients with Mild Cognitive Impairment

**DOI:** 10.3390/biomedicines13102447

**Published:** 2025-10-08

**Authors:** Laura Danesin, Giorgia D’Este, Rita Barresi, Elena Piazzalunga, Agnese Di Garbo, Andreina Giustiniani, Carlo Semenza, Gabriella Bottini, Massimiliano Oliveri, Francesca Burgio

**Affiliations:** 1 IRCCS San Camillo Hospital, 30126 Venezia, Italy; giorgia.deste@hsancamillo.it (G.D.); rita.barresi@hsancamillo.it (R.B.); elena.piazzalunga@hsancamillo.it (E.P.); francesca.burgio@hsancamillo.it (F.B.); 2 Scuola Universitaria Superiore IUSS, 27100 Pavia, Italy; 3 Restorative Neurotechnologies Srl, 90143 Palermo, Italy; agnese.digarbo@restorativeneurotechnologies.com; 4 IRCCS Centro Neurolesi “Bonino Pulejo”, 98124 Messina, Italy; andri.giustiniani@gmail.com; 5 Department of Neurosciences, University of Padova, 35131 Padova, Italy; carlo.semenza@unipd.it; 6 Department of Brain and Behavioral Science, University of Pavia, 27100 Pavia, Italy; gabriella.bottini@unipv.it; 7 ASST GOM Niguarda, 20162 Milan, Italy; 8 NeuroMI, 20126 Milan, Italy; 9 Department of Biomedicine, Neurosciences and Advanced Diagnostics (BiND), University of Palermo, 90133 Palermo, Italy; massimiliano.oliveri@unipa.it

**Keywords:** cognitive dysfunction, prismatic adaptation, cognitive training, Alzheimer’s disease, Parkinson’s disease, brain-derived neurotrophic factor, biomarkers, brain plasticity

## Abstract

**Background/Objectives**: This study evaluated a novel rehabilitation tool that combines prismatic adaptation (PA) and cognitive training through serious games (SGs) in patients with mild cognitive impairment (MCI) due to prodromal Alzheimer’s dementia or consequent to Parkinson’s disease. While non-pharmacological interventions have been shown to improve cognition or delay dementia onset, their underlying neurobiological mechanisms, such as brain plasticity, remain unclear. Leveraging studies suggesting neuromodulatory effects of PA, we investigated whether the combined PA+SGs treatment could influence plasticity-related mechanisms, assessed through brain-derived neurotrophic factor (BDNF) serum levels, compared to cognitive training with only SGs and standard cognitive rehabilitation (SCR). **Methods**: Twenty three MCI patients were randomized into three intervention groups: PA+SGs (experimental group), SG-only (control group), and SCR (control group), completing ten treatment sessions. Patients underwent neuropsychological assessments and blood sampling pre- and post-treatment. **Results**: At baseline, demographic, clinical, and biological characteristics were comparable across groups. Post-treatment, though differences from control groups were not statistically significant, the PA+SGs group showed significant within-group improvements in memory, with trend-level changes also in executive function and visuospatial abilities, which, however, did not reach the significance threshold. Notably, only the PA+SGs group exhibited increased BDNF levels, which positively correlated with memory and language performance. **Conclusions**: Our findings suggest that combining PA with cognitive training may enhance cognitive functioning in MCI patients, yielding results comparable to SCR. Furthermore, PA appears to enhance neuroplasticity mechanisms that may support the behavioral improvements observed in cognitive training. Future research should validate these findings and further explore the relationship between cognitive impairment and its rehabilitation, while also considering the underlying neurobiological mechanisms.

## 1. Introduction

Mild cognitive impairment (MCI) is defined as a state of cognitive decline that is more prominent than expected for an individual’s age and educational level, and that does not significantly interfere with daily activities [1]. Historically, the term MCI has been applied to identify a prodromal stage of dementia in patients with Alzheimer’s disease (AD), but it has since been broadened to include cognitive impairment due to other etiologies, such as dementia with Lewy body, Parkinson’s disease (PD), vascular dementia, or frontotemporal dementia [2]. Overall, the prevalence of MCI in older adults is estimated to range from 5% to 30%, with an annual incidence rate of 22.6 per 1000 persons [3]. Although MCI may be a stable condition [4], it represents one of the main risk factors for dementia [5]. Approximately 11% to 33% of cases of MCI convert to dementia within two years [6], with patients showing memory deficits (amnestic MCI) being twice as likely to progress to dementia as non-amnestic ones. Moreover, patients with MCI may show multi-domain cognitive deficits, affecting, for instance, language [7], calculation [8,9], attention, and executive function [10] in addition to memory, which can subsequently impact daily tasks such as decision-making and financial independence [9]. Overall, given the ongoing growth of the elderly population [11], MCI is a matter of significant public health concern.

To date, there are no recognized pharmacological therapies for MCI, and behavioral interventions have been designed to improve cognitive performance or help individuals compensate for cognitive difficulties [12]. Among these, cognitive rehabilitation approaches have shown promising results [13,14], especially when relying on the use of new technologies [15]. In particular, in recent years, new programs for cognitive training were developed using a gamification approach. Serious games (SGs) are defined as digital games created with the intention of achieving outcomes beyond entertainment alone [16]. SGs are used to overcome some of the limitations of classical cognitive rehabilitation, as they can increase the patient’s motivation and involvement in the rehabilitation process [17]. Moreover, they allow therapists to easily personalize treatment to the patient’s needs, thanks, for instance, to a dynamic adaptation of difficulty parameters to the patient’s performance [18]. In addition, several studies have been exploring the possibility of combining cognitive training with non-invasive brain stimulation (NIBS) techniques, such as transcranial magnetic stimulation (TMS) or transcranial direct current stimulation (tDCS). Although these techniques act differently on cortical excitability, they can both induce synaptic plasticity through long-term potentiation or long-term depression [19,20], ultimately inducing changes that can support the reorganization of the brain networks involved in the impaired cognitive function [21,22]. In this regard, the combination of NIBS with cognitive training has shown promising results in various populations [23,24]. Recently, some studies have suggested that prismatic adaptation (PA), a visuomotor procedure that induces a shift in the visual field using prismatic lenses, may increase cortical excitability in the frontoparietal network of the hemisphere ipsilateral to the prism deviation [25], with effects similar to those of tDCS [26].

PA typically involves performing visuomotor tasks, such as pointing toward a target under different experimental conditions that require updating an individual’s visuomotor coordinates to maintain pointing accuracy [27]. PA has been widely used in research and clinical practice to treat spatial neglect [28], but it also shows broader applicability in sensorimotor and cognitive disorders [29,30]. From a behavioral perspective, PA-based interventions have mostly involved healthy individuals or stroke patients and have been linked to improvements in spatial attention [31]. However, some studies also highlight effects beyond spatial domains, as PA seems to influence verbal and spatial memory [32], as well as working memory, attention, and inhibitory control [33,34,35]. These findings are further supported by neuroimaging studies, indicating that PA may modulate functional connectivity within attentional and frontoparietal networks, particularly involving the intraparietal sulcus and superior parietal lobule, with additional activation of temporo-parietal and occipital regions [36,37,38]. At the network level, PA may also promote brain reorganization by decoupling the default mode network from attentional systems, potentially underlying its rapid and widespread behavioral effects [36]. Overall, evidence suggests that PA facilitates neuroplasticity across distributed brain networks, supporting the recovery of spatial functions while also improving broader cognitive domains.

Moreover, a recent pilot study on stroke patients combining PA with cognitive training suggested that PA could prime cortical excitability in order to make it more responsive to cognitive training, thus supporting its positive effects [35].

Nevertheless, PA cognitive effects in patients with MCI have been scarcely studied [39,40], and the mechanisms underlying the effects of cognitive rehabilitation, whether used alone or in combination with other techniques (e.g., PA), are still not fully understood. In other words, it is not clear to what extent the rehabilitation may act only on the symptomatic component (i.e., cognitive impairment) or also on its underlying processes, such as neurodegenerative or neuroplastic ones. To shed light on this issue, some studies have suggested the use of the brain-derived neurotrophic factor (BDNF) as a potential marker for the effectiveness of interventions [41]. BDNF is a neurotrophin secreted by neurons in the brain, crucial for maintaining both cognitive and motor functions [42]. It supports neurons and promotes synaptic plasticity through long-term potentiation [43]. Higher BDNF levels at baseline have been associated with decreased odds of developing dementia [44]. Moreover, increased BDNF serum levels have been linked to the effectiveness of non-pharmacological interventions in ameliorating cognitive performance across various populations [45,46]. However, studies focusing on patients with MCI have yielded inconsistent results [47,48].

The aims of the present exploratory study were manifold. Firstly, we aimed to investigate the association between BDNF serum levels and the degree of cognitive impairment in a cohort of patients with MCI due to different underlying aetiologies, specifically AD or PD. Moreover, we aimed to explore the effectiveness of different rehabilitative approaches on the diverse cognitive impairments associated with the disease and their potential to promote brain plasticity. To these aims, we compared changes in cognitive performance and BDNF serum levels between patients who underwent traditional cognitive rehabilitation, SG-based cognitive training, or SG-based cognitive training combined with PA. In particular, we hypothesized that PA could be a useful tool to enhance the beneficial effects of cognitive training and promote plastic changes in patients with MCI.

## 2. Materials and Methods

The present study is part of a single-blind RCT clinical trial registered on Clinicaltrials.gov (NCT05826626).

### 2.1. Participants

Study participants were recruited among patients admitted to IRCCS San Camillo Hospital or referred for clinical screening due to suspected cognitive impairment from September 2021 to August 2023. All eligible participants had already been diagnosed with AD or PD by external neurologists before participating in the study. Inclusion criteria were: (i) aged 40-85 years old; (ii) diagnosis of MCI [1,49]; (iii) preserved use of at least one hand; (iv) absence of psychiatric illnesses and/or comorbidity with other neurological pathologies (e.g., acquired brain injuries, cerebrovascular pathology, neuroinflammatory diseases, multiple sclerosis); and (v) ability to provide informed consent. A randomization sequence with a 1:1:1 design was applied to randomly allocate eligible participants into three groups: combined cognitive training using PA and SGs (PA+SG, experimental group), cognitive training using only SGs (SG-only, control group), and conventional cognitive rehabilitation (SCR, control group). Before and after the treatment, patients underwent cognitive and clinical assessments, as well as blood sample acquisition. All evaluations were performed by experienced and trained neuropsychologists. Blood sample acquisitions were performed by trained nurses. Biological analyses were performed blind. All participants took part in the study voluntarily and gave informed consent according to the Declaration of Helsinki. The study was approved by the Ethics Committee of Venice and IRCCS San Camillo Hospital (Venice, Italy), reference number 2021.12.

### 2.2. Cognitive and Clinical Assessment

Each patient completed a comprehensive neuropsychological evaluation before and immediately after the treatment. The assessment included measures of attention, executive functions, memory, visuospatial abilities, and language. In particular, the following tests were administered: (i) Mini-Mental State Examination (MMSE) for general cognitive functioning; (ii) Trail Making Test (TMT) for attention; (iii) Stroop test, Clock Drawing Test (CDT), digit and spatial span backward, and phonological fluencies for executive functions; (iv) digit and spatial span forward, Rey Auditory Verbal Learning Test (RAVLT), prose memory, and recall of the Rey–Osterrieth Complex Figure (ROCF-recall) for learning and memory domain; (v) copy of the Rey–Osterrieth Complex Figure (ROCF-copy) for the visuospatial and visuoconstructive domain; and (vi) semantic fluencies for the language domain.

Lastly, psychological concerns were assessed using the State-Trait Anxiety Inventory (STAI-Y) and Beck Depression Inventory-II (BDI-II).

### 2.3. Intervention

All groups completed ten 30-min sessions in two weeks (five sessions/week).

The first group (PA+SG) underwent the experimental treatment using MindLenses Professional (Restorative Neurotechnologies, Palermo, Italy, 2021, https://www.restorativeneurotechnologies.com/en/cognitive-rehabilitation-medical-device, 20 July 2025), a new tool that allows coupling the digital implementation of PA with the administration of SGs [35]. Specifically, the PA procedure was performed in the first five minutes of each session, followed by approximately twenty minutes of digital cognitive exercises (SGs). The pointing task was performed on a tablet under three conditions: pre-exposure, exposure, and post-exposure. The pre-exposure and the post-exposure conditions counted 30 trials each (10 for each target position), and the exposure condition counted 90 trials (30 for each target position). In the exposure condition, patients performed the pointing task while wearing prisms, inducing a 10° shift in the visual field. The direction of the shift (right vs. left deviation) was randomized across patients, as no clear effects of different deviations are reported in the literature in this population. Therefore, half of the patients completed the treatment with right-deviating prisms and the other half with left-deviating ones. Patients were instructed to keep their index finger at the sternum level and to place the fingertip on the target at a fast but comfortable speed. Pointing movements were executed with the right hand. The sight of the hand and the arm was allowed for the whole duration of the procedure (concurrent exposure procedure). The tablet resolution was 1200 × 2000 pixels, with a pixel density of 224 ppi. Spatial displacement of the pointing movements was recorded automatically by the tablet in terms of pixels. Immediately after PA, seven SGs were administered every day in the same order. The games were designed to tackle various components of attention, executive function, and language. In detail, the games addressed visual search, sustained and alternate attention, planning, inhibition, verbal and abstract reasoning, working memory, short-term memory, and calculation [35].

In the second group (SG-only), the same digital exercises of the MindLenses protocol were administered without first performing the PA procedure.

Lastly, patients in the SCR groups completed ten sessions of conventional cognitive training using the Erica^®^ software (Giunti Psychometrics, Firenze, Italy, 2013, https://www.giuntipsy.it/erica-esercizi-di-riabilitazione-cognitiva, (accessed on 20 July 2025)).

### 2.4. Blood Samples Collection and Serum Isolation

Blood samples were drawn from patients before and after completing the treatment and collected in BD Vacutainer^®^ SST™ II Advance vials (#366882A, BD Vacutainer, Becton Dickinson and Company, Franklin Lakes, NJ, USA). Samples were left to coagulate for 30 min at room temperature (RT) and then successively centrifuged for 15 min at 1500× *g*. The serum was then divided into aliquots and stored at −80 °C before use.

### 2.5. Brian-Derived Neurotrophic Factor (BDNF) Assay

Serum isolated from patients’ blood was thawed on ice prior to analysis. BDNF concentration in the serum was tested with the ChemiKine BDNF Sandwich ELISA Kit (#CYT306, CHEMICON International, Inc., Temecula, CA, USA) according to the manufacturer’s instructions. Briefly, samples were diluted (1:100) in sample diluent, added to the plate along with the standard samples, and run in duplicate. After overnight incubation at 4 °C and washing with washing solution, incubation with the primary antibody was performed for 2 h at room temperature (RT). Wells were then incubated with the horseradish peroxidase-conjugated secondary antibody for 1h at RT. After washing, tetramethylbenzidine solution was added for 15 min at RT; the reaction was stopped with the stop solution to allow visualization. Absorbance was measured at 450 nm with the Infinite F50 Plus plate reader (TECAN Trading AG, Männedorf, Switzerland).

### 2.6. Statistical Analysis

As a preliminary step, scores on cognitive tests were corrected for age, education, and gender using normative data for the Italian population. The Shapiro–Wilk test was then used to assess the data distribution. As the majority of variables exhibited deviations from normality, non-parametric tests were used for further analyses. We examined differences in demographic variables between groups using the Kruskal–Wallis test for continuous variables or the chi-square test for nominal ones. One-way non-parametric ANOVAs using the Kruskal–Wallis test were then conducted to investigate baseline differences between groups at the cognitive and clinical evaluations.

As an exploratory step, paired-sample t-tests using the Wilcoxon signed-rank test were computed separately for each group to explore within-group changes in cognitive performance after the treatment. Afterward, to investigate the presence of differences between groups in the pre- vs. post-treatment changes, non-parametric ANOVAs using the Kruskal–Wallis test were applied to delta scores, computed for each measure by subtracting pre-treatment scores from post-treatment ones. Post hoc tests using Mann–Whitney U were then applied to explore differences in changes in pre- vs. post-treatment cognitive performance between the experimental and the two control groups. Bonferroni’s correction for multiple comparisons was then applied to control for inflated type I error rates. Effect sizes were computed following the method described by Rosenthal [50].

Concerning BDNF levels, preliminary correlations were performed to investigate whether baseline BDNF levels and post-treatment changes may be associated with demographic characteristics, such as age and education. Similarly, non-parametric one-way ANOVAs using the Mann–Whitney U test were conducted to explore the association between BDNF levels and categorical variables, such as gender, and clinical characteristics, such as MCI pathology (AD or PD), and MCI type (single vs. multiple domain). Correlation analyses were then performed using Spearman’s rho to investigate the baseline association between cognitive scores and BDNF serum levels and to explore if changes at the cognitive level could be associated with changes in BDNF depending on the type of rehabilitation received.

All statistical analyses were conducted with SPSS 23 (SPSS, 2015).

## 3. Results

### 3.1. Sociodemographic and Baseline Characteristics of the Sample

**Figure 1:**
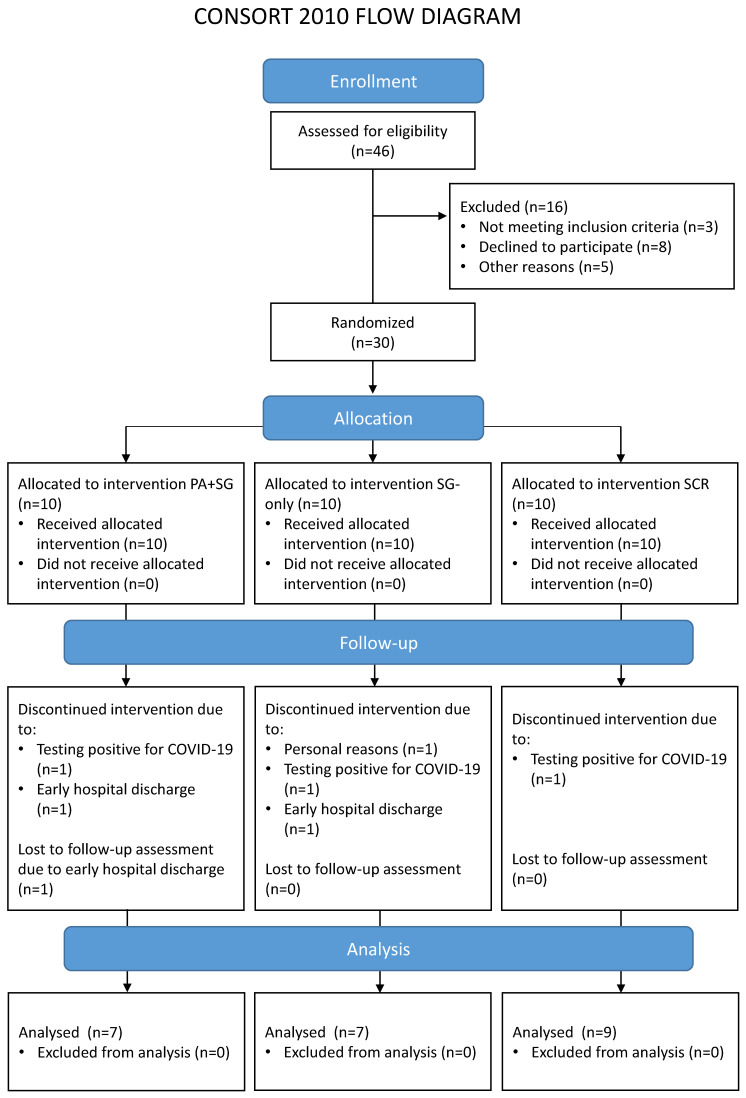
Flow diagram of the participants involved in the different phases of the study. The enrolled participants were randomly assigned to one of the three interventions: a combination of prismatic adaptation and serious games (PA+SG—experimental group), an intervention with only serious games (SG-only—control group), or standard cognitive rehabilitation (SCR—control group).

A total of 46 potential participants were contacted and screened for eligibility, of whom 30 were enrolled in the study. Main reasons for exclusion concerned: not meeting the inclusion criteria (e.g., no evidence of MCI at the cognitive assessment, n = 1; evidence of moderate/severe cognitive impairments suggestive of progression to dementia, n = 2); declining to participate in the study (e.g., not interested in the study, n = 8); and other reasons related to logistic or personal problems that would prevent treatment adherence (n = 5). Seven enrolled patients did not complete the rehabilitation for reasons unrelated to the study (tested positive for COVID-19, n = 3; early discharge, n = 3; or personal reasons, n = 1). Therefore, a total of 23 patients were included in the analysis. No statistical differences emerged when comparing drop-outs and included patients in the baseline characteristics (see Table S1 in the Supplementary Materials). Table 1 reports the sociodemographic characteristics of the final sample, separately for each treatment group. Figure 1 shows the Consolidated Standards of Reporting Trials (CONSORT) flowchart, which outlines participant flow through the study and reasons for drop-out.

All groups had similar age (*p* = 0.672), educational level (*p* = 0.072), and gender distribution (*p* = 0.906). Concerning patients’ cognitive profiles (Table 2), at baseline, no significant differences emerged between groups in all the administered neuropsychological tests (all *p* > 0.050). Notably, most of the sample showed difficulties in more than one cognitive domain (multi-domain MCI). Moreover, no significant differences were observed in baseline BDNF serum levels between the three intervention groups (H = 3.297, *p* = 0.192).

Exploratory analyses were performed to investigate whether baseline BDNF serum levels could be associated with the demographic or clinical characteristics of the sample. No significant correlations were observed with age (rho = −0.224, *p* = 0.484) or educational levels (rho = −0.331, *p* = 0.143). Similarly, baseline BDNF levels were not significantly associated with gender (U = 41.00, *p* = 0.456) or other clinical characteristics of the sample (MCI pathology: U = 48.00, *p* = 0.670; MCI type: U = 47.00, *p* = 0.913). Conversely, a significant positive correlation emerged at baseline between BDNF levels and scores the phonological fluency test (rho = 0.509, *p* = 0.019).

### 3.2. Post-Treatment Cognitive Changes

**Figure 2:**
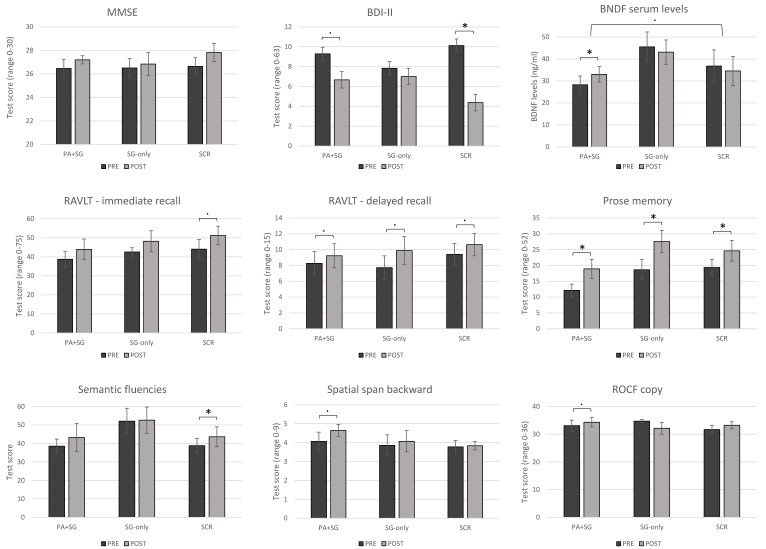
Pre- and post-treatment changes across the three intervention groups (PA+SG: combination of prismatic adaptation and serious games–experimental group; SG-only: rehabilitation with only serious games–control group; SCR: standard cognitive rehabilitation–control group). Significant differences between measures (*p* < 0.05) are depicted using asterisks, while trends toward significance are shown using dots (*p* < 0.07).

Concerning within-group changes between the two timepoints, participants in the experimental group (PA+SG) obtained significantly increased scores in the prose memory test (z = −2.197, *p* = 0.028) with medium–large effect sizes (r = −0.59). Moreover, trends of improvements with medium effect sizes were observed in the delayed recall of the RAVLT (z = −1.841, *p* = 0.066, r = −0.49), the copy of ROCF (z = −1.826, *p* = 0.068, r = −0.49), spatial span back (z = −1.947, *p* = 0.052, r = −0.52), and BDI-II scores (z = −1.897, *p* = 0.058, r = −0.55). The SG-only group showed significant improvements in prose memory (z = −2.207, *p* = 0.027), with a large effect size (r = −0.64), and a trend in RAVLT-delayed recall (z = −1.841, *p* = 0.066, r = −0.53). Conversely, the group who underwent standard cognitive rehabilitation obtained medium–large increased scores in semantic fluency (z = −2.033, *p* = 0.042, r = −0.48) and prose memory (z = −2.201, *p* = 0.028, r = −0.59), alongside a significant reduction in scores in the BDI-II scale (z = −2.383, *p* = 0.017, r = −0.60). Moreover, the SCR group showed trends of improvements in RAVLT-immediate recall (z = −1.955, *p* = 0.051, r = −0.46) and RAVLT-delayed recall (z = −1.955, *p* = 0.053, r = −0.46). Figure 2 illustrates the pre- and post-treatment cognitive performance across the three intervention groups for tests that showed significant within-group changes or trends toward significance. Additional figures showing pre- and post-treatment cognitive performance for tests without significant differences are included in the Supplementary Materials (Figure S1).

When analyzing the presence of between-group differences in changes pre- vs. post-treatment at cognitive and clinical levels, no significant differences were observed.

### 3.3. Association Between Changes in BDNF Serum Levels, Demographic Characteristics, and Cognitive Performances

**Figure 3:**
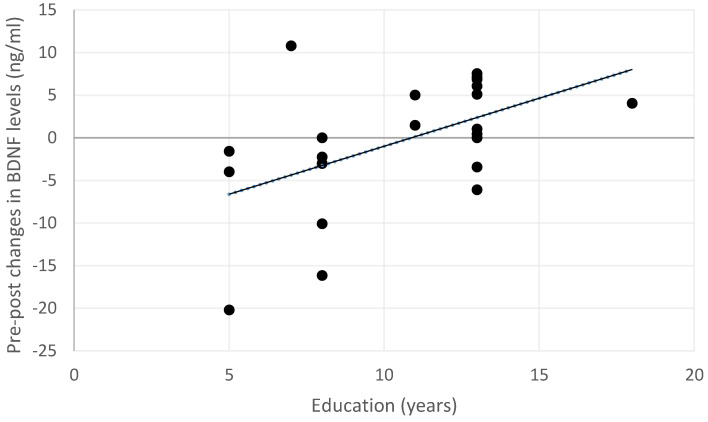
Relationship between changes in BDNF serum levels and educational levels across the entire sample. Positive changes indicate an increase in BDNF levels, while negative changes indicate a decrease in the post-treatment measurement.

Exploratory analyses were performed to investigate whether changes in BDNF serum levels may depend on the demographic or clinical characteristics of the sample. At the post-treatment acquisition, changes in BDNF levels after the treatment were not associated with age (rho = −0.175, *p* = 0.587), gender (U = 31.00, *p* = 0.140), or clinical characteristics (MCI pathology: U = 47.00, *p* = 0.619; MCI type: U = 29.00, *p* = 0.149). Instead, a positive correlation was observed between increased BDNF levels and educational level (rho = 0.478, *p* = 0.028; see also Figure 3).

Concerning within-group changes between the two timepoints, participants in the experimental group (PA+SGs) showed increased BDNF serum levels (z = −2.201, *p* = 0.028) with a large effect size (r = −0.64). When analyzing the presence of between-group differences in changes pre- vs. post-treatment, a trend toward significance emerged for changes in BDNF levels (X^2^ = 5.570, *p* = 0.062). In the post hoc analysis, no significant differences were observed after Bonferroni’s correction for multiple comparisons. However, a trend toward significance emerged when comparing changes in BDNF levels in the experimental group (PA+SGs) versus the SCR group (U = 9.00, *p* = 0.068, r = −0.55).

**Figure 4:**
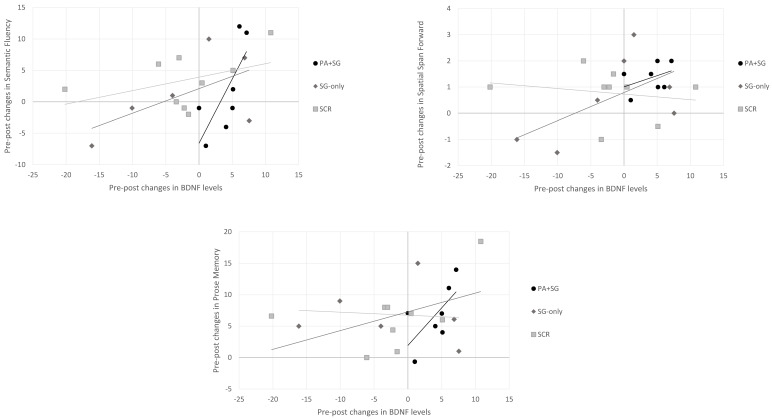
Relationship between changes in BDNF serum levels and changes in cognitive tests across the three intervention groups (PA+SG: combination of prismatic adaptation and serious games–experimental group; SG-only: rehabilitation with only serious games–control group; and SCR: standard cognitive rehabilitation–control group). Positive changes indicate an increase in BDNF levels or cognitive performance, while negative changes indicate a decrease in the post-treatment assessment.

Concerning the association between changes at the biological and cognitive levels separately for each treatment group (Figure 4), positive correlations were observed between increased BDNF levels and scores on the semantic fluency (rho = 0.943, *p* = 0.005), prose memory (rho = 0.829, *p* = 0.042), and spatial span forward tests (rho = 0.820, *p* = 0.046) in the experimental group (PA+SGs). Similarly, in the SG-only group, a positive association was found between scores on the spatial span forward test (rho = 0.943, *p* = 0.005) and BDNF levels, whereas no significant correlation emerged in the SCR group.

## 4. Discussion

The present study aimed to explore the effects of a new cognitive training tool (MindLenses Professional), which combines prismatic adaptation (PA) and serious games (SGs), in a cohort of patients with mild cognitive impairment (MCI). Specifically, we aimed to investigate whether this new tool could improve cognitive performance, at least with effects similar to programs routinely used in clinical practice. Moreover, as previous studies have suggested that PA may induce neuromodulatory effects on cortical excitability [25,26], we also explored whether changes at the behavioral level may reflect changes in plasticity-related mechanisms, as assessed through BDNF serum levels.

When examining within-group changes, our preliminary findings suggest that treatment with MindLenses may significantly enhance cognitive functioning in patients with MCI, especially concerning verbal memory performance. Furthermore, the exploratory analysis revealed some more subtle changes in other areas, such as spatial working memory and visuospatial skills, which did not reach the significance threshold (*p* < 0.05) and therefore should be interpreted with caution. Similarly, a slight decrease in depressive symptoms was observed. Notably, the experimental group did not show statistically significant differences in cognitive changes compared to those undergoing standard cognitive rehabilitation, suggesting that MindLenses may have effects similar to previously validated programs used in clinical practice. These results are consistent with earlier research on healthy individuals and other clinical populations. Improvements in attention after PA are well documented in the literature [29,51], with several studies also showing changes in functional brain connectivity, primarily involving the dorsal attention network and the default mode network [36,52]. Consequently, we can hypothesize that combining cognitive rehabilitation with PA may work through a bottom-up process, where PA may directly boost attentional performance. Since attention is crucial for executing other cognitive tasks properly [53,54], increased attention might also lead to improvements in other cognitive functions. Recent studies by Turriziani and colleagues have shown increased memory and executive function in healthy individuals following PA, with left-deviating prisms enhancing verbal memory and mental flexibility, and right-deviating prisms improving spatial memory [32,34]. These effects have also been observed in patients with acquired brain injuries, such as stroke survivors. Studies using the combined PA+SG treatment report improvements in memory, visuospatial skills, attention, and executive functions, along with increased functional independence and reduced anxiety levels [35,55]. Therefore, PA may help boost attentional resources for subsequent rehabilitative exercises, which could explain the broader cognitive gains observed. Overall, these findings suggest that MindLenses could be an effective tool for cognitive rehabilitation across different conditions, including neurodegenerative and cerebrovascular disorders.

Moreover, the results of this study contribute to the existing body of evidence by investigating potential neurobiological mechanisms underlying behavioral changes. In particular, we found that patients in the experimental group exhibited a significant increase in BDNF serum levels after treatment, which was not observed in either of the two control groups. Notably, on a qualitative level, 100% of patients in the PA+SG group showed increased BDNF levels after treatment, while this percentage was lower in the other two groups (50% in SGs and 66.6% in SCR), indicating that the neuromodulation exerted by MindLenses had a significant effect on plasticity. The finding of increased brain plasticity after neuromodulation aligns with a previous study in patients with PD, which reported increased BDNF levels following stimulation of fronto-central regions using anodal tDCS [56].

The exploration of the association between BDNF levels and cognitive functioning reveals interesting interactions. A positive correlation emerged at baseline with scores on the phonological fluency test, which serves as a proxy for mental flexibility. Of note, verbal fluency tests have been shown to possess high sensitivity in detecting MCI [57]. Furthermore, a significant association was found between changes in BDNF and educational levels. In particular, education may be indicative of an individual’s overall cognitive reserve, although other factors, such as occupation or type of leisure activities, are also involved [58]. Cognitive reserve refers to the set of mechanisms that the brain can enact “to cope with damage by using pre-existing cognitive processes or enlisting compensatory strategies” [58], and it has been linked to the flexibility of synaptic reorganization after brain damage [59]. Similarly, higher cognitive reserve has been identified as a protective factor against the development of dementia in patients with MCI [60,61]. Overall, these associations suggest an interplay between neuroplasticity and cognitive functioning in patients with MCI. On the one hand, patients with a higher degree of impairment at baseline may also exhibit reduced plastic resources; on the other hand, higher cognitive reserve may constitute a positive prognostic factor, as patients with higher plastic resources could benefit more from the neuromodulation induced by prisms.

Lastly, in the experimental group, increased BDNF levels were associated with improved performance in language and memory functioning. This finding aligns with previous studies linking BDNF expression with hippocampal functioning (see [62] for a review), suggesting that non-pharmacological interventions, especially aerobic ones, may increase BDNF peripheral levels and memory functioning while reducing depressive symptoms and hippocampal atrophy.

Nevertheless, our findings must be interpreted cautiously, especially when considering its possible clinical implications. For instance, one may speculate that higher BDNF levels post-treatment may translate into higher retention of the rehabilitation effects or slower disease progression; however, no causal implications can be drawn from our results, and further studies are needed to explore these hypotheses.

A primary limitation of the present exploratory study was the limited sample size (n = 23), which limits statistical power and increases the risk of both Type I and Type II errors. As such, the present findings should be interpreted as exploratory and cannot be generalized until validated in larger, adequately powered cohorts. Our power analysis indicated that the current sample size is sufficient to detect only large effects (f = 0.40), while detecting medium effects (f = 0.25) with 80% power would require at least 42 participants. These estimates provide useful guidance for the design of future studies aimed at confirming and extending our results in the neurodegenerative population. Additionally, the limited sample size prevented more detailed analyses. For example, due to insufficient power, we could not stratify patients based on their underlying MCI pathology (AD vs. PD) or MCI type (amnestic/non-amnestic, single/multi-domain). Future studies should replicate these findings with larger samples to validate the observed effects and trends and to investigate how clinical factors may influence cognitive recovery after cognitive training or mediate neuroplasticity mechanisms, possibly also directly comparing the effects on different patient populations (e.g., MCI-AD vs. MCI-PD). Moreover, widening the sample size may also allow for a more in-depth investigation of the effect of the combined PA+SG treatment, depending on the applied prism deviation. On this line, another limitation of the present study concerns its single-blind nature, as participants were aware of the type of treatment they were receiving. Future studies should consider the adoption of specific designs to minimize expectancy bias, such as the use of prismatic goggles with sham deviation. Additional research is also needed to investigate the possible effects of PA on brain functional connectivity in MCI patients and address whether PA-induced changes in the BDNF peripheral levels could be linked to functional reorganization of specific brain networks, which may underlie cognitive recovery. Lastly, regarding biological analyses, our results may be confounded by external factors that were not measured for the purpose of the study. For instance, the peripheral concentration of BNDF may depend on non-modifiable factors, such as the genetic polymorphisms [63] or sex [64], as well as modifiable behaviors like diet or physical activity [64,65]. Future studies should consider these factors in their design and attempt to control for potential confounding variables, while also investigating the potential role of other biomarkers in the effects of cognitive rehabilitation for MCI patients.

## 5. Conclusions

In conclusion, this exploratory study provides preliminary evidence that cognitive training combined with PA may enhance cognitive functioning and promote neuroplasticity in patients with MCI. Of note, the absence of significant differences compared with standard cognitive rehabilitation highlights that the combined intervention may yield benefits comparable to established programs. Importantly, the consistent increase in BDNF observed only in the PA+SG group suggests that this intervention may stimulate neuroplastic mechanisms that underlie cognitive recovery. These findings align with prior research demonstrating PA’s capacity to modulate brain networks and facilitate broader cognitive recovery. Therefore, from a clinical perspective, our findings highlight the potential of integrating PA into cognitive rehabilitation to offer a biologically grounded strategy to support cognitive recovery.

Despite these promising results, several challenges remain. The small sample size and single-blind design restrict generalizability, and future research should validate these findings in larger, adequately powered cohorts. Stratification by MCI subtype and etiology, adoption of additional control conditions to minimize bias, and integration of multimodal biomarkers (e.g., functional connectivity, genetic factors) may clarify the mechanisms of action of behavioral interventions. Additionally, longitudinal designs are needed to determine whether increases in BDNF may be associated with longer retention of cognitive improvements or predict slower progression toward dementia. Overall, this study contributes to the growing evidence that combining neuromodulation with cognitive training holds promise for advancing precision rehabilitation in MCI.

## Figures and Tables

**Table 1:** Sociodemographic characteristics of the patients enrolled in the study. Mean, standard deviation, number of patients, and percentages are reported. Results of the Kruskal–Wallis (H) or the chi-square test (X2) are reported for continuous and categorical variables, respectively.

Characteristics	Whole Sample (n = 23)	PA+SG (n = 7)	SG-Only (n = 7)	SCR (n = 9)	H/X^2^ (*p*-Value)
Age, years (SD)	73.91 (8.00)	72.43 (11.12)	72.14 (8.47)	76.44 (4.19)	0.795 (0.672)
Education, years (SD)	10.43 (3.09)	12.71 (0.76)	9.43 (2.99)	9.44 (3.54)	5.253 (0.072)
Gender, n. females (%)	9 (39.1)	2 (28.6)	3 (42.9)	4 (44.4)	0.475 (0.789)
MCI pathology, n. AD (%)	11 (47.8)	3 (42.9)	3 (42.9)	5 (55.6)	1.168 (0.558)
MCI type, n. single-domain (%)	8 (34.8)	2 (28.6)	3 (42.9)	3 (33.3)	0.329 (0.849)

**Table 2:** Scores at the cognitive and clinical evaluation across the three treatment groups. Means and standard deviations of corrected scores are reported for each test/clinical scale and BDNF serum levels (ng/mL).

Domain	Test/Scale	PA+SG		SG		SCR	
		T0	T1	T0	T1	T0	T1
General cognitive functioning	MMSE	26.46 (2.01)	27.20 (0.91)	26.50 (2.10)	26.84 (2.56)	26.64 (2.21)	27.82 (2.34)
Attention	TMT-A	44.57 (21.95)	35.29 (38.61)	37.17 (30.24)	34.83 (26.81)	42.78 (34.91)	37.33 (31.69)
	TMT-B	113.0 (73.45)	134.0 (91.75)	71.8 (114.03)	95.6 (78.02)	92.25 (76.96)	109.5 (98.59)
Executive function	Phonological Fluency	33.90 (15.86)	38.87 (25.30)	38.13 (8.93)	37.73 (12.45)	32.46 (11.53)	35.56 (9.40)
	Stroop test-n. errors	0.57 (1.77)	0.321 (0.59)	3.58 (7.01)	3.21 (5.51)	0.78 (1.35)	2.056 (3.48)
	Stroop test-time	27.07 (28.84)	15.00 (6.64)	12.08 (10.76)	18.68 (16.20)	23.42 (16.16)	20.89 (15.13)
	CDT	8.14 (2.27)	8.21 (1.50)	7.20 (4.09)	8.33 (3.60)	8.19 (2.31)	9.06 (0.77)
	Digit span backward	4.23 (1.11)	3.79 (0.86)	4.00 (0.82)	4.14 (0.90)	4.00 (0.97)	3.67 (1.12)
	Spatial span backward	4.07 (1.27)	4.64 (0.85)	3.86 (1.46)	4.07 (1.48)	3.78 (1.00)	3.83 (0.66)
Memory	Digit span forward	5.57 (0.73)	5.43 (1.54)	5.93 (1.64)	5.29 (0.81)	4.78 (1.28)	5.17 (1.20)
	Spatial span forward	4.29 (0.93)	4.71 (0.81)	4.29 (1.41)	4.86 (1.57)	3.94 (0.88)	4.72 (0.51)
	RAVLT-immediate recall	38.73 (11.15)	43.97 (14.27)	42.58 (5.79)	48.20 (14.61)	44.04 (15.15)	51.26 (14.73)
	RAVLT-delayed recall	8.27 (3.95)	9.23 (4.03)	7.72 (3.97)	9.89 (4.65)	9.41 (4.13)	10.63 (4.25)
	ROCF-recall	14.18 (8.28)	17.00 (9.40)	16.04 (7.24)	17.50 (8.98)	16.06 (4.85)	18.46 (7.49)
	Prose memory						
Language	Semantic fluency	33.86 (17.85)	35.57 (17.86)	38.33 (8.76)	39.50 (14.75)	36.67 (11.15)	40.11 (10.88)
Visuospatial Abilities	ROCF-copy	33.07 (5.29)	34.32 (4.55)	34.71 (1.50)	32.12 (5.69)	31.67 (4.47)	33.21 (3.86)
Clinical scales	STAI-Y2	34.86 (8.23)	38.00 (6.26)	36.33 (10.46)	34.00 (5.57)	40.67 (11.14)	40.78 (12.03)
	BDI-II	9.29 (3.90)	6.67 (3.88)	7.83 (4.26)	7.00 (6.38)	10.13 (4.52)	4.38 (4.24)
	IADL	6.71 (1.70)	7.17 (1.17)	5.00 (2.16)	5.80 (1.48)	5.75 (2.06)	5.83 (1.60)
Biological variables	BDNF	28.27 (10.51)	33.01 (9.30)	45.54 (18.02)	43.15 (14.67)	36.82 (21.98)	34.57 (19.73)

MMSE: Mini-Mental State Examination; TMT: Trail Making Test; CDT: Clock Drawing Test; RAVLT: Rey Auditory Verbal Learning Test; ROCF: Rey–Osterrieth Complex Figure; STAI: State-Trait Anxiety Inventory; BDI: Beck Depression Inventory; IADL: Instrumental Activities of Daily Living; BDNF: Brain-Derived Neurotrophic Factor.

## Data Availability

Data supporting the findings of this study are available on request from the corresponding author. Data are not publicly available due to privacy or ethical restrictions.

## Supplementary Materials

The following supporting information can be downloaded at: https://www.mdpi.com/article/10.3390/biomedicines13102447/s1, Table S1: Demographic characteristics of patients who completed the rehabilitation program and of drop-outs. Figure S1. Pre-post treatment changes across the three intervention groups (PA+SG: combination of prismatic adaptation and serious games—experimental group; SG-only: rehabilitation with only serious games—control group; SCR: standard cognitive rehabilitation—control group). All changes concerning measures reported in the figure did not reach significance levels (*p* > 0.05).

## Abbreviations

The following abbreviations are used in this manuscript:

PA	Prismatic Adaptation
SGs	Serious Games
MCI	Mild Cognitive Impairment
BDNF	Brain-Derived Neurotrophic Factor
SCR	Standard Cognitive Rehabilitation
AD	Alzheimer’s Disease
PD	Parkinson’s Disease
NIBS	Non-Invasive Brain Stimulation
TMS	Transcranial Magnetic Stimulation
tDCS	Transcranial Direct Current Stimulation
MMSE	Mini-Mental State Examination
TMT	Trail Making Test
CDT	Clock Drawing Test
RAVLT	Rey Auditory Verbal Learning Test
ROCF	Rey–Osterrieth Complex Figure
STAI-Y	State-Trait Anxiety Inventory
BDI-II	Beck Depression Inventory
