# Supplementary material for: Preliminary Evidence of Biological and Cognitive Efficacy of Prismatic Adaptation Combined with Cognitive Training on Patients with Mild Cognitive Impairment
Source: Biomedicines. 2025 Oct 8;13(10):2447. doi: 10.3390/biomedicines13102447 (PMC12561499; doi:10.3390/biomedicines13102447)

Title: Preliminary Evidence of Biological and Cognitive Efficacy of Prismatic Adaptation Combined with Cognitive Training on Patients with Mild Cognitive Impairment

Authors:
Danesin Laura, D'Este Giorgia, Barresi Rita, Piazzalunga Elena, Di Garbo Agnese, Giustiniani Andreina, Semenza Carlo, Bottini Gabriella, Oliveri Massimiliano, Burgio Francesca

Journal: Biomedicines

Corresponding author
Laura Danesin, PhD, IRCCS San Camillo Hospital, via Alberoni 70, 30126, Venice, Italy
laura.danesin@hsancamillo.it

Supplementary materials:

Table S1. Demographic characteristics of patients who completed the rehabilitation program and of drop-outs.

	Whole sample (n=30)	Drop-outs patients (n=7)	Included patients (n=23)	U / X^2 (p-value)
Age, y (sd)	74.03 (7.17)	74.43 (3.73)	73.91 (8.00)	70.5 (.631)
Education, y (sd)	10.00 (3.38)	7.86 (2.27)	10.65 (3.43)	41.5 (.054)
Gender, n. F (%)	10 (33.3)	1 (10.0)	9 (39.1)	1.491 (.372)
MMSE (sd)	26.93 (2.66)	26.99 (4.47)	27.14 (2.17)	54.5 (.976)

Figure S1. Pre-post treatment changes across the three intervention groups (PA+SG: combination of prismatic adaptation and serious games - experimental group; SG-only: rehabilitation with only serious games - control group; SCR: standard cognitive rehabilitation - control group). All changes concerning measures reported in the figure did not reach significance levels (p>0.05).

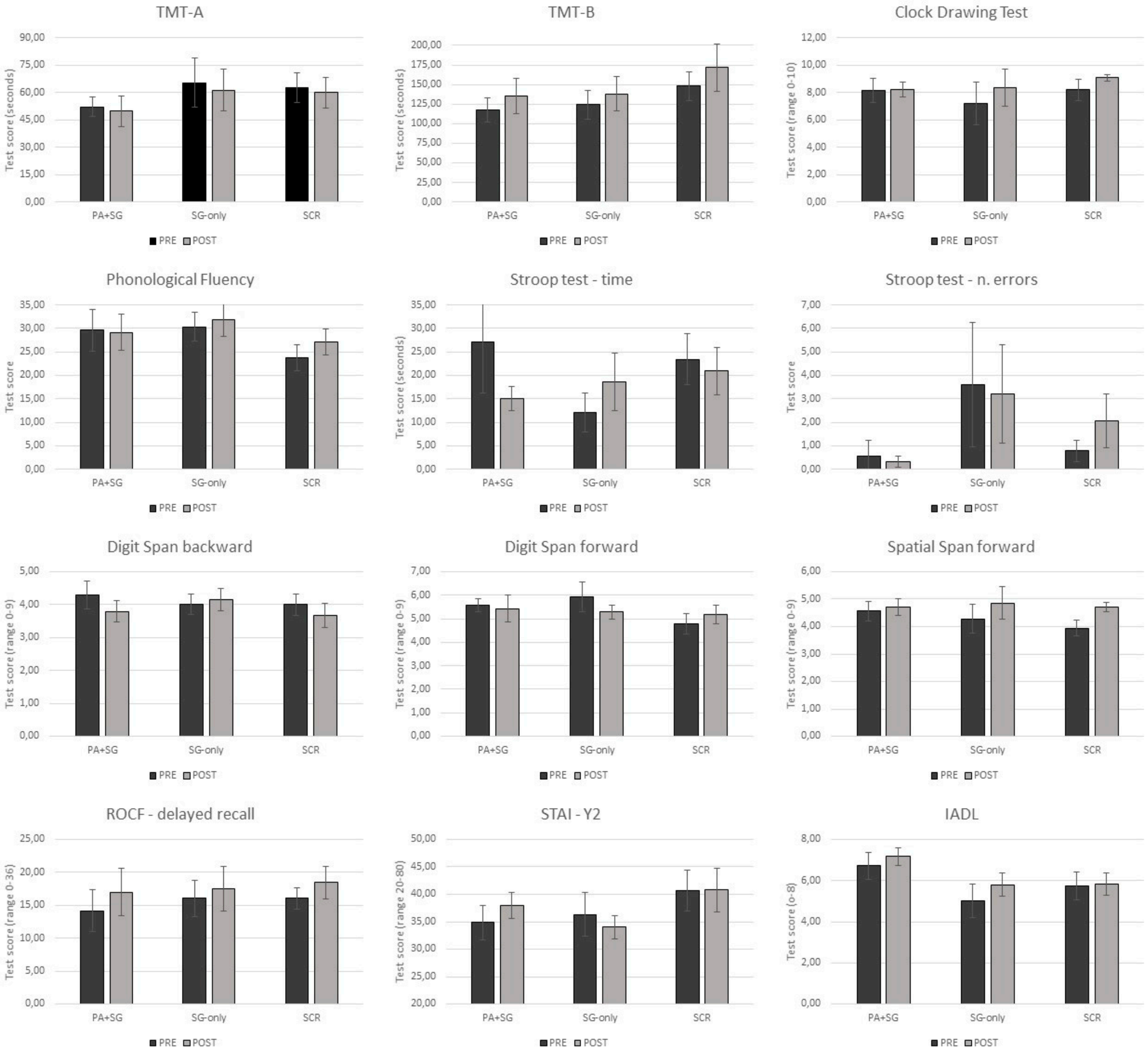

Supplement: Supplementary file 1 [file biomedicines-13-02447-s001.zip › biomedicines-3800307-supplementary.pdf]
